# An integrated care pathway for maternal and childcare: evidence from Ethiopia, Tanzania, and Uganda

**DOI:** 10.1093/eurpub/ckac129.393

**Published:** 2022-10-25

**Authors:** I Corazza, P Belardi, M Bonciani, F Manenti, D Abebe, S Santini, G Azzimonti, J Nsubuga, G Dall'Oglio, M Vainieri

**Affiliations:** 1 Health and Management Laboratory, Sant'Anna School of Advanced Studies, Pisa, Italy; 2 Doctors with Africa CUAMM, Padua, Italy

## Abstract

Performance monitoring and evaluation are key to quality improvement in maternal and child healthcare in Sub-Saharan Africa. This study presents the experience of designing and implementing bottom-up and integrated performance evaluation tools for care pathway to monitor and manage maternity healthcare services. The research project involved four health districts and relative reference hospitals, supported by the NGO Doctors with Africa CUAMM in Ethiopia, Tanzania, and Uganda. The maternal and childcare pathway developed consists of 23 indicators, calculated at hospital and district level, relating to pregnancy, childbirth and first year of life phases. The authors developed staves and performance maps, as graphical representation tools, to display longitudinally integrated health services provision performance along the continuum of care. Substantial variation was observed between the phases of each maternal and childcare pathway and across the care pathways of the different analysed settings. The most impressive results across the four settings are: 1) regarding pregnancy phase, that women tend to attend more than four antenatal classes, still with a quite high drop-out rate, and are largely tested for syphilis, 2) with respect to childbirth, that there are varying percentage levels in terms of C-sections, episiotomies and peri/intra-partum asphyxia cases, and 3) as it regards first year of life, there emerges scope for improvement considering the vaccination coverages attained for pentavalent, measles and polio vaccinations. Thanks to the collaboration with local managers and health professionals, the maternal and childcare pathway allowed to monitor the changes in the quality of maternity services provided within the analysed contexts. The benchmarking approach encouraged local professionals to learn from other settings. The use of such tool allowed the development of targeted quality improvement actions, shared among all involved stakeholders.

**Key messages:**

• In collaboration with local professionals, we designed and implemented an integrated pathway for maternal and childcare, covering the phases of pregnancy, childbirth and first year of life.

• Benchmarking performance results encouraged collaboration among professionals and allowed the identification of actions to improve the provision of maternal and childcare services.

